# Experimental Study on Tunnel Bottom Deformation Trend in Gently Inclined Layered Shale Based on the Energy Index

**DOI:** 10.3390/ma16237433

**Published:** 2023-11-29

**Authors:** Binke Chen, Yinjun Tan, Yuan Deng, Zheng Liu, Wei Meng

**Affiliations:** 1 School of Civil Engineering, Chongqing Jiaotong University, Chongqing 400074, China; becker91910315@gmail.com (B.C.); mw@cqjtu.edu.cn (W.M.); 2 School of Civil Engineering, Southwest Jiaotong University, Chengdu 610031, China; yinjuntan_3639@163.com

**Keywords:** energy index, gently inclined layered shale, uniaxial compression test, discriminating indicator, tunnel bottom deformation tendency

## Abstract

Influenced by the anisotropy and water-softening characteristics of gently inclined layered shale, many tunnels have encountered bottom deformation issues during construction and operation, which severely impact the safety of tunnel structures. The energy evolution law during rock deformation and damage can provide support for the assessment and prediction of structure deformation. However, most studies have been conducted on enstatite, granite, and sandstone with limited research on shale. In this study, both conventional and single-cyclic loading-and-unloading uniaxial compression tests were conducted on shale specimens with varying dip angles of the structural plane (*Dφ*) and water content (*Wc*) in addressing the most typical layered shale in the Chaoyang Tunnel. The energy evolution features of rock samples at each stage of the tests were analyzed to determine the discriminating indicator (*SC*) for tunnel bottom deformation tendency. The indicator was based on the elastic strain energy (*U^e^_i_*) and the post-peak dissipation energy (*U^d^_i_*). The results demonstrated that the *Dφ* and *Wc* directly affected the energy storage and dissipation process of rock specimens, which in turn enabled them to exhibit different damage evolution features. The *U^e^_i_* and the total input energy (*U^l^_i_*) satisfied a linear relationship, which was determined by the *Dφ* and *Wc* of rock specimens. The energy evolution-based indicator *SC* can accurately characterize the bottom deformation of the tunnel constructed in a gently inclined layered shale stratum. The findings can offer a scientific foundation for rational evaluation of the structure deformation of tunnels under construction.

## 1. Introduction

The gently inclined layered rock mass generally exhibits typical orthogonal anisotropy or transverse isotropy, and its stability is controlled by the structural plane of rock blocks [1]. The mechanical properties of gently inclined layered rock mass demonstrate strong anisotropy, inhomogeneity, and discontinuity, and show spatial–temporal variabilities of destruction and deformation under the coupling action of multiple fields [2]. The stability of the tunnel face in this type of rock mass usually faces various problems. Numerous tunnels built and under construction in gently inclined layered rock mass are frequently experiencing severe hazards caused by the tunnel bottom deformation [3]. Therefore, it is of great significance for reasonable assessment and prediction of the bottom deformation of tunnels built in gently inclined layered rock mass.

The deformation and failure processes of rock mass involve the energy input, accumulation, dissipation, and release [4,5]. The loading-induced deformation and damage of rock mass are primarily the outcome of the combined processes of energy release and dissipation [6,7,8], where the energy dissipation mainly contributes to inducing rock damage, leading to material property deterioration and strength loss, while energy release is generally responsible for the sudden damage of the rock mass [9]. Study of the energy evolution law throughout the process of rock deformation and damage, as well as the establishment of the correlation between the damage pattern and energy density can clarify the deformation characteristics during the tunnel construction [10,11].

By incorporating parameters such as elastic energy and dissipation energy, the energy transformation patterns during the rock mass damage process can be more accurately and efficiently described [12,13,14]. Wasantha et al. [15] analyzed the mechanical features and energy release traits of layered rock mass throughout the uniaxial compression tests using the acoustic emission monitoring. Meng et al. [16] analyzed the sandstone’s energy evolution law under circumstances of uniaxial cyclic stress and unloading. Li et al. [17] proved that there was a close correlation between the deformational damage of rocks and the proportion of dissipated energy through mechanical tests. Chen et al. [18,19] investigated the energy change characteristics of kyanite and granite and concluded that there were differences in the energy release law of rocks at different strains. By conducting the uniaxial cyclic loading tests, Liu et al. [20] established a constitutive model to describe the failure characteristics of sandy mudstones and siltstones based on the features of energy dissipation. Steffler et al. [21] developed a finite element model based on the moiré interferometry method to describe the energy variations and crack propagation characteristics of geological materials under compressive stress.

More recently, a number of more accurate models have been developed as a result of revisions and refinements to existing research results. Liu et al. [22] established a stress–strain model that can accurately describe the damage law of rock samples on the basis of Weibull distribution theory and the introduction of energy release rate. Mahanta et al. [23] investigated the influence of different strain rates on the fracture toughness and energy release rate of shale. Gong et al. [24] found that unloading confining pressure does not affect the rock failure mode but induces a strength reduction effect in the rock. By conducting uniaxial compression tests on granite and red sandstone, Du et al. [25] clarified the correlations among the hard rock energy, the energy storage coefficient corresponding to peak-strength strain, and the residual elastic energy index. He et al. [26] investigated the energy evolution laws of sandstone and granite from both macroscopic and microscopic perspectives based on multistage cyclic loading. Luo et al. [27] proposed the calculation methods for elastic strain energy and dissipative strain energy corresponding to the peak point under multiple stress degrees. Li et al. [28] investigated the impact of initial flaws on the progression of rock damage and devised a damage model for fractured rocks by considering the energy dissipation.

However, most of the available studies have been conducted on enstatite, granite, and sandstone, and there are very limited studies on shale. In addition, the scientific achievements mainly focus on the influence of stress path or strain rate on the deformation and damage process under loading, and the correlation between energy feature parameter and tunnel deformation in practical conditions has not yet been established. For the tectonic characteristics of the gently inclined layered shale, the structural plane angle and water content significantly affect its deformation pattern, which are the parameters that must be considered for investigating its mechanical properties.

In view of this, the loading-and-unloading tests under uniaxial compression were performed on a gently inclined layered shale specimen with different *D_φ_* and *W_c_*, and its dissipated energy and elastic strain energy evolution laws under different conditions were analyzed. By introducing the elastic compression energy storage coefficient (*S*) and brittle ductility index (*C*), a new energy-based method for predicting bottom deformation of a tunnel in gently inclined layered shale strata was developed, and its accuracy was verified with the field monitoring data. The findings in this study can provide a new idea for the assessment and prediction of structure deformation of tunnels in gently inclined layered rock mass.

## 2. Project Overview

The Chaoyang Tunnel of the Qiannan Passenger Dedicated Line is a tunnel segment located between Dushan South and Libo in Guizhou Province [29]. It has a total length of 12,734 m and a maximum burial depth of approximately 432 m. The longitudinal slope of the route is a single downhill gradient, with a descent of 25‰ at the entrance end and a slope length of 12,598 m, and a descent of 22.7‰ at the exit end with a slope length of 136 m.

**Figure 1:**
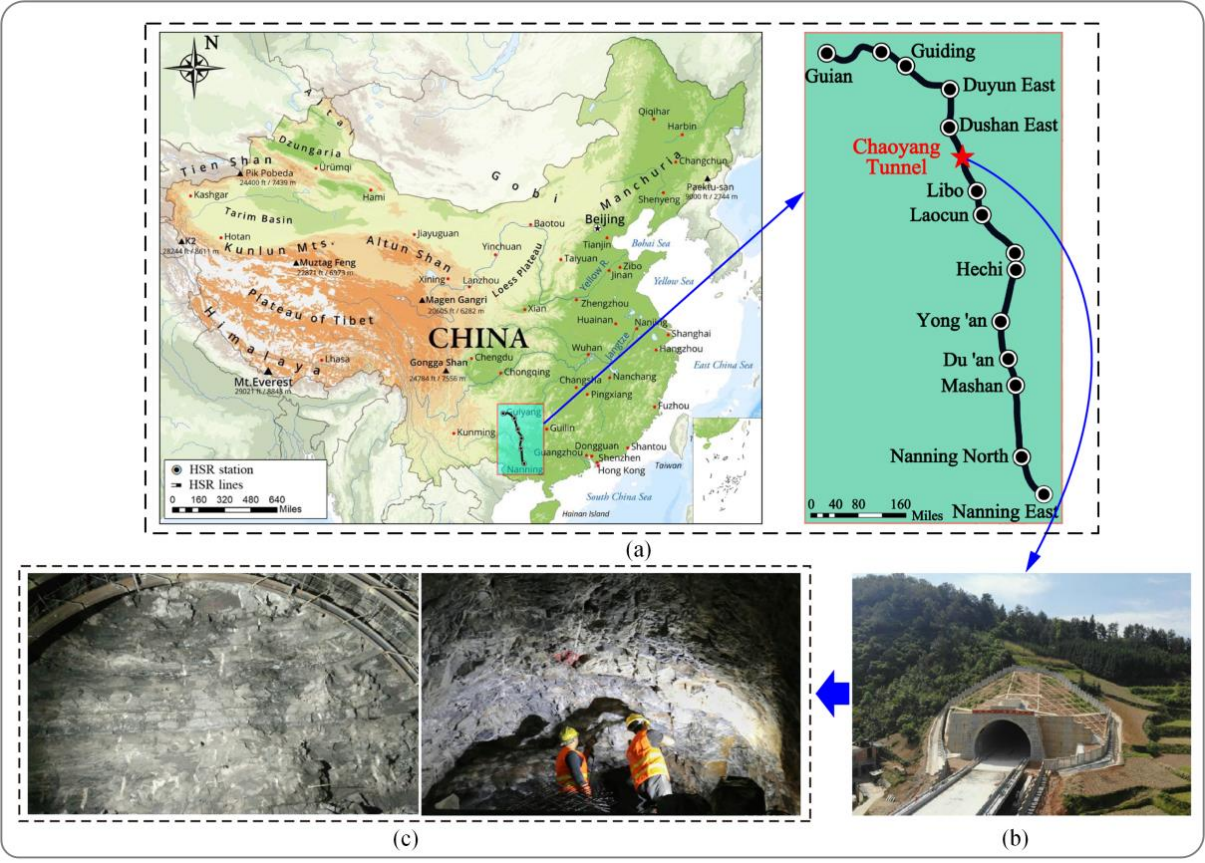
Topographic and geomorphological sketch of the Chaoyang Tunnel [29]: (**a**) Location of the project; (**b**) Real view of the Chaoyang Tunnel; (**c**) Geological formations of tunnel face.

The study area features a mid-low mountainous karst hill terrain, with slopes ranging from 20 to 55 degrees, and locally forming steep cliffs. The gently inclined section of the tunnel’s surrounding rock has a total length of 4.27 km, with burial depths ranging from approximately 200 to 370 m. Based on regional data and onsite surveys, three developed faults were identified within the survey area, namely, the Diebu 1# normal fault, Diebu 1# reverse fault, and Chaoyang reverse fault. The groundwater exhibits diverse types, with corrosiveness to concrete due to sulfuric acid, and the degree of soil erosion is classified as H1. The tunnel predominantly traverses the Lower Carboniferous Datang Period (C1d1) old profile, with lithology mainly composed of argillaceous limestone, interspersed with shale, carbonaceous shale, and sandstone. The thickness of the geological layers is moderate, occasionally with thin interlayers, characterized by clay cementation. Shale layers are generally thin, displaying a laminated structure with leaf-like and flake-like features. Figure 1 depicts the geography and geomorphology of the Chaoyang Tunnel.

## 3. Experimental Program

### 3.1. Specimen Preparation

In the geological survey process of the Chaoyang Tunnel, the gently inclined layered shale was identified as the most typical rock type in this region, with inclinations mainly around 0°, 15°, and 30°. Additionally, due to the water-softening characteristics of this rock type, the moisture content significantly influences the mechanical behavior and failure characteristics of rock samples, which is an essential factor to be considered. Therefore, this study conducted experimental research by setting three moisture content levels and three structural plane inclinations for the gently inclined layered shale specimens.

**Figure 2:**
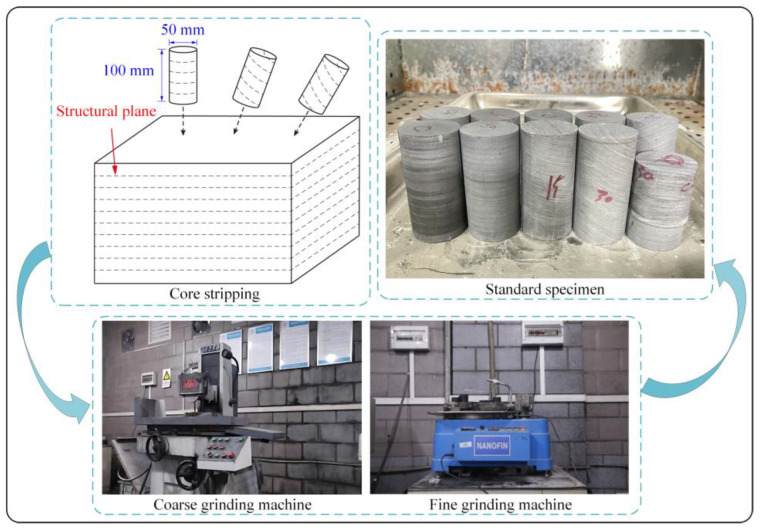
Specimen preparation process.

With reference to the regulation of International Society for Rock Mechanics (ISRM) [30], the gently inclined layered shale blocks taken from Chaoyang Tunnel were fabricated for the physical and mechanical properties tests. The fabrication and preparation processes are shown in Figure 2.

After coring, rough cutting, fine cutting, and polishing, cylindrical rock specimens measuring 100 mm high and 50 mm in diameter were obtained. The specimens must satisfy the dimensional error of less than ±0.5 mm and the end parallelism error of less than ±0.02 mm [31].

The following treatments were carried out to obtain rock specimens with different water contents:

(1) The standard specimen was directly wrapped with plastic wrap as a natural specimen (with a water content of 2.4%).
(2) The standard specimen was placed in a dryer, and the drying temperature was kept constant at 105 °C. After drying for 24 h, it was taken out to obtain the dried specimen (with a water content 0%).
(3) To obtain a fully saturated specimen (with a water content of 5.3%), the partially dried specimen was placed in a water saturator under vacuum conditions for a duration of 24 h.

### 3.2. Test Method

In this study, we conducted both conventional uniaxial compression tests and single-cyclic loading-and-unloading uniaxial compression tests. The latter involved the process of loading, unloading, reloading, and inducing damage. The peak strength and damage characteristics of the gently inclined layered shale specimens were analyzed by conducting the conventional uniaxial compression test. Furthermore, the influence of *D_φ_* and *W_c_* on the energy evolution of the specimens was investigated through a single-cyclic loading-and-unloading uniaxial compression test.

#### 3.2.1. Uniaxial Compression Test

**Figure 3:**
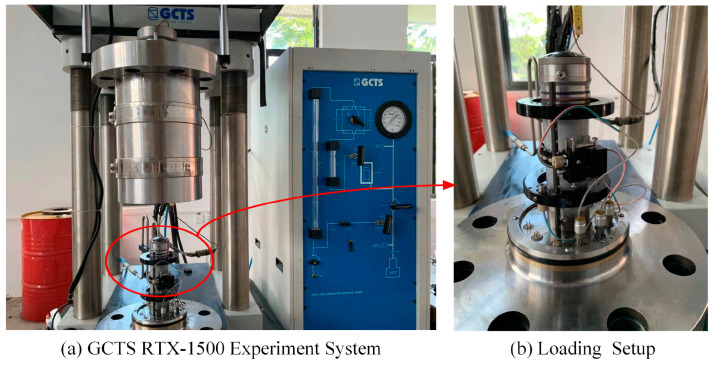
Specimen preparation process.

The RTX-1500 Rock Mechanics Test System produced by the U.S. GCTS company was utilized to perform uniaxial compression tests (Figure 3). It can withstand axial forces up to 1500 kN and confining pressures up to 140 MPa. The specimen was pre-loaded to 2 MPa at a rate of 0.1 MPa/s before the loading. Once the columnar steel seat above the specimen came into contact with the upper indenter of the press, the specimen was subjected to a loading rate of 0.05 MPa/s until failure. The damage features and peak strength of the specimen were recorded during the test.

#### 3.2.2. Loading–Unloading Test under Uniaxial Compression Condition

**Figure 4:**
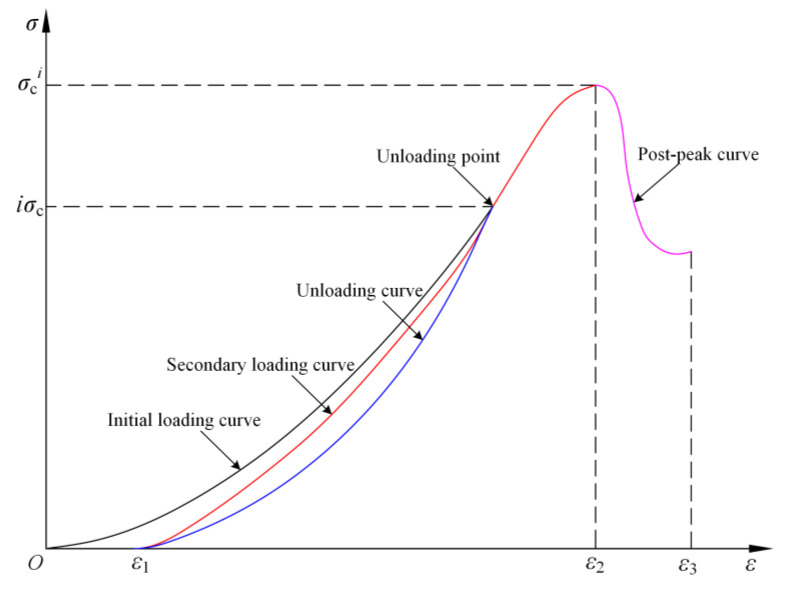
Stress–strain curves for loading–unloading tests under uniaxial compression conditions.

Conventional uniaxial compression tests were used to obtain stress–strain curves and peak strengths. Subsequently, single-cyclic loading-and-unloading tests were conducted at various unloading levels. The loading equipment (i.e., GCTS RTX-1500 Rock Mechanics Experiment System) and pre-loading method were consistent with those of the conventional uniaxial compression tests. Different from the conventional uniaxial compression tests, these tests were set with different unloading levels *i*. The specimens were loaded by the stress-controlled mode (0.05 MPa/s) to *iσ_c_*, and then the loading was unloaded to 0. After the unloading was completed, the loading was applied to the specimen again until the specimen was completely damaged. To minimize the impact of sudden destabilization on the test findings, the secondary loading stage was controlled at a loading rate of 0.05 mm/min. The peak strength *σ_c_^i^* during the secondary loading stage was extracted at the end of the test, and the loading-and-unloading stress paths are depicted in Figure 4.

The specimens were set up with three *W_c_* values, while three *D_φ_* values were set up under each *W_c_* scenario. A total of nine groups of specimens were prepared for the single-cyclic loading-and-unloading uniaxial compression test to study the energy evolution laws of gently inclined layered shale with different *D_φ_* and *W_c_*. The test scenarios and serial numbers are shown in Table 1.

## 4. Results and Discussion

### 4.1. Uniaxial Compression Test

#### 4.1.1. Failure Modes

**Figure 5:**
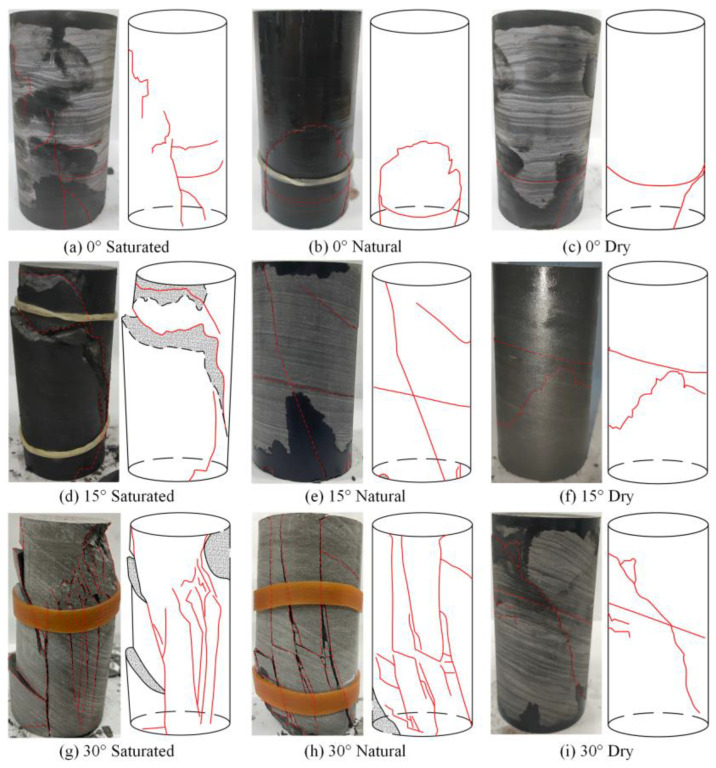
Failure modes of specimens (Black dashed line: rock mass exfoliation cross section; Red solid line: crack development path).

The compression-induced macroscopic crack expansion patterns of the gently inclined layered shale specimens with different values of *D_φ_* and *W_c_* are shown in Figure 5. It was observed that there is a strong correlation between the crack expansion pattern of the specimen and the *D_φ_* and *W_c_* during uniaxial compression. The results also revealed that there are primarily three damage patterns, which are shear-slip failure, tension-split failure, and tension-shear composite failure.

The specimens under uniaxial compression conditions with the structural plane angle of 0° all experienced obvious circumferential cracks on the surfaces, accompanied by a series of cracks developing approximately along the vertical direction. The specimens presented tensile failure characteristics along the axial loading direction, which is mainly tensile splitting failure along the structural plane. At the *D_φ_* of 15°, the specimens with different water contents all yielded cracks through the structural plane, with obvious characteristics of shear-slip failure along the structural plane, and accompanied by some vertically developed cracks. This part of the cracks was mainly caused by tension splitting failure, and the overall failure was a tension-shear composite failure dominated by the shear-slip damage along the structural plane. At the *D_φ_* of 30°, the specimens suffered from different degrees of through cracks from the upper left to the lower right direction, exhibiting typical tension-shear composite failure characteristics. The main reason for this phenomenon is the significant deviation between the loading direction and the structural plane. As a result, the structural plane experienced relative slip within the specimen when subjected to vertical loading. Meanwhile, the number of surface cracks increased with higher water content at the same *D_φ_*, and the approximate loading direction fractures continued to grow and eventually penetrated the circumferential cracks. Spalling of rock fragments occured on the surface of some specimens with more serious damage, which is most pronounced in the saturated specimens with the *D_φ_* of 30°.

#### 4.1.2. Variation Characteristics of Peak Stress and Elastic Modulus

**Figure 6:**
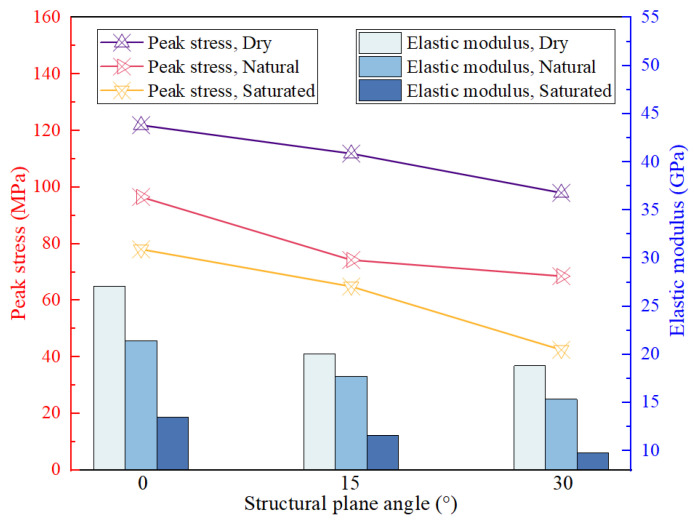
Test results of peak stress and elastic modulus.

Each group of specimens’ elastic modulus and peak strength is shown in Figure 6. The increases in the *D_φ_* and *W_c_* adversely affected the specimens’ elastic modulus and peak strength, and this adverse effect was more significant under the combined effect of anisotropy and water-softening. The elastic modulus and peak strength of the dried specimen were maximum at the *D_φ_* of 0°. As the *D_φ_* rose to 30° and the *W_c_* reached saturation condition, the specimens’ peak strength and elastic modulus decreased by 65.2% and 63.9%, respectively, which are the lowest values of all the specimens.

The strength characterization values of specimens with the same *D_φ_* decreased with the increase in the *W_c_*, where the decrease in the modulus of elasticity intensified with increasing *W_c_*. Taking the specimen with the *D_φ_* of 0° as an example, the modulus of elasticity decreased by 20.9% when the *W_c_* changes from dry to natural. When the *W_c_* changes from natural to saturated, the modulus of elasticity decreased by 37.3%. This is because the shale possesses the water-softening property, and when water penetrates the pores of shale, it will lead to the shale absorbing the water and expanding. Consequently, the bond between the internal particles will be weakened and become relatively soft, resulting in deformation and cracking. For specimens with the same *W_c_*, the *D_φ_* and the strength characterization values also satisfied the negative correlation. As an example, the modulus of elasticity and peak stress of the dried specimen decreased by 26.0% and 8.2%, respectively, when the *D_φ_* increases from 0° to 15°. When the *D_φ_* increases from 15° to 30°, the modulus of elasticity and peak stress decreased by 6.3% and 12.4%, respectively.

### 4.2. Energy Evolution Characteristics of Gently Dipping Bedded Shale

#### 4.2.1. Energy Evolution Theory of Rock Damage Processes

Under the assumption that there is no heat exchange with the external environment during the deformation process of a rock element subjected to external forces, according to the first law of thermodynamics, it is evident that the energy input *U* from the external load can be partitioned into elastic energy *U^e^* and dissipation energy *U^d^* [31]. Thus, the total energy *U*, along with *U^e^* and *U^d^*, must satisfy the following relationship:

(1) $$U=U^{d}+U^{e}$$

where *U^e^* is the energy stored within the rock during the loading, which can be released and recovered after unloading, and *U^d^* is the energy dissipated during the loading due to the internal damage (continuous generation, expansion, and penetration of microfractures) and the specimen’s plastic deformation.

**Figure 7:**
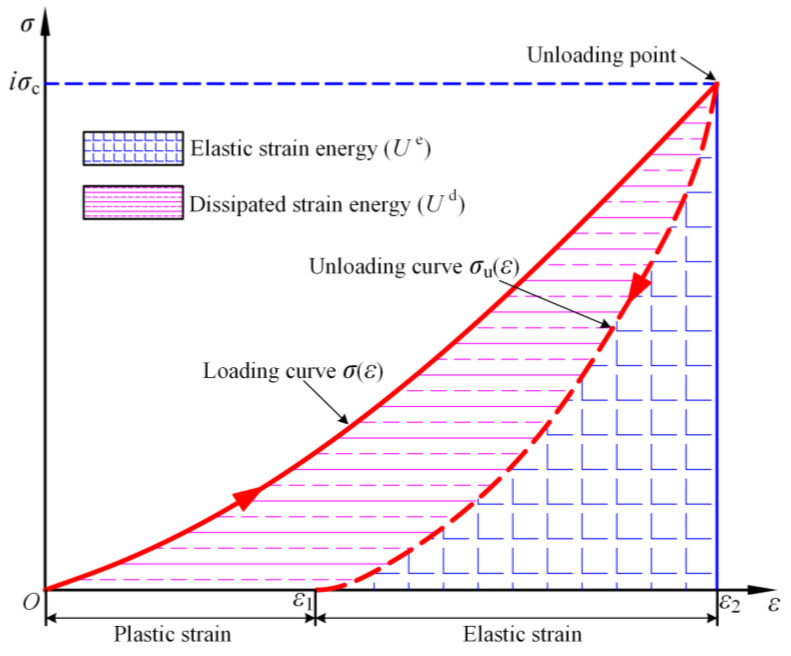
Energy relations in rock units under uniaxial compression.

Based on the unloading segment of the load-displacement curve [32], the elastic energy stored within the rock sample during single-cyclic loading-and-unloading uniaxial compression test can be calculated. Figure 7 illustrates the stress–strain curves of the rock element at the unloading point *iσ_c_* under uniaxial compression conditions. The area under the loading curve at the unloading point represents the total input energy *U*, the area under the unloading curve is the elastic energy *U^e^*, and the dissipated energy *U^d^* is the area between the loading curve *σ*(*ε*) and the unloading curve *σ*_u_(*ε*).

During the loading and unloading, the *U*, *U^e^*, and *U^d^* per unit of rock can be calculated according to Equations (2)–(4):

(2) $$U=\int_{0}^{\varepsilon_{2}}\sigma (\varepsilon )d\varepsilon$$

(3) $$U^{e}=\int_{\varepsilon_{1}}^{\varepsilon_{2}}\sigma_{u}(\varepsilon )d\varepsilon$$

(4) $$U^{d}=U-U^{e}=\int_{0}^{\varepsilon_{2}}\sigma (\varepsilon )d\varepsilon -\int_{\varepsilon_{1}}^{\varepsilon_{2}}\sigma_{u}(\varepsilon )d\varepsilon$$

where *ε*_1_ corresponds to the strain in the loading direction when the specimen is unloaded along the unloading curve to a load of 0; *ε*_2_ corresponds to the strain in the loading direction at the unloading point; *σ*(*ε*) is a function of the rock specimen’s stress–strain curve during the loading stage; *σ*_u_(*ε*) is a function of the rock specimen’s stress–strain curve during the unloading stage.

#### 4.2.2. Analysis of Energy Evolution Characteristics

1. The energy evolution laws at various unloading degrees

**Figure 8:**
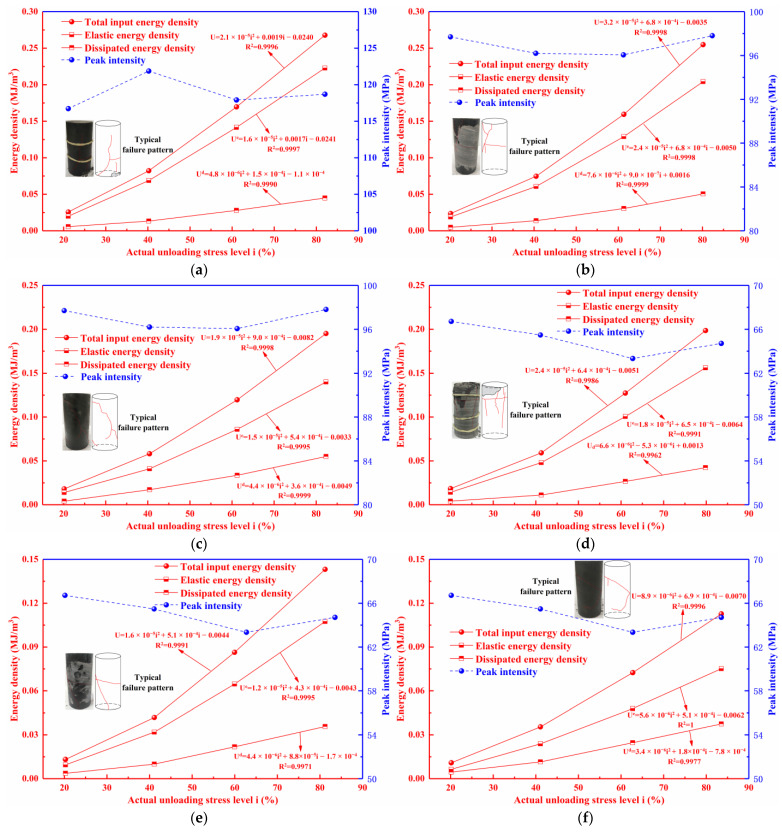

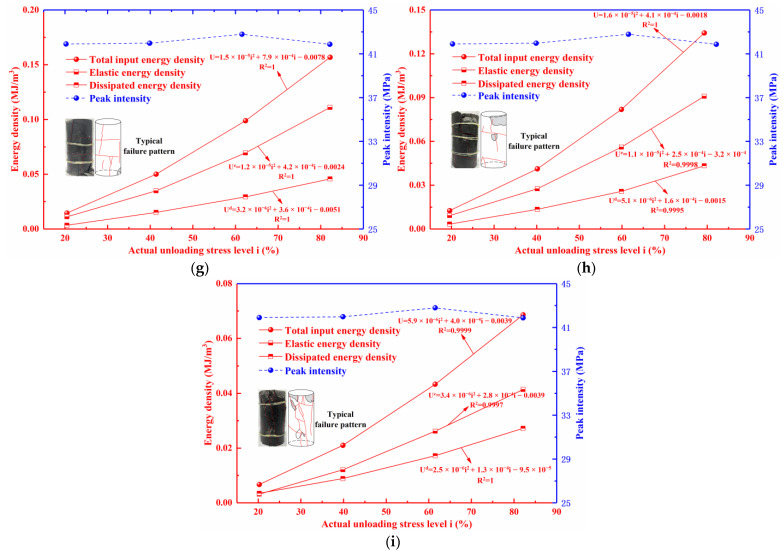
Energy evolution curves of specimens with different working conditions: (**a**) Dry, 0°; (**b**) Dry, 15°; (**c**) Dry, 30°; (**d**) Natural, 0°; (**e**) Natural, 15°; (**f**) Natural, 30°; (**g**) Saturated, 0°; (**h**) Saturated, 15°; (**i**) Saturated, 30°.

The whole process from the initial deformation to the final damage of the rock in the uniaxial compression test can be regarded as an energy transformation process including the input, storage, dissipation, and release of energy. Before the peak stress, the load-induced work was mainly transformed into elastic energy for storage, and part of the energy was transformed into dissipated energy due to damage or plastic deformation. The actual unloading level *i*, total input energy (*U^l^_i_*), elastic energy (*U^e^_i_*), and dissipated energy (*U^d^_i_*) for each test scenario can be obtained by calculating the area under the stress–strain curve. On this basis, the specimens’ energy evolution laws in different test scenarios were obtained, and the equation fitting the specimens’ energy density to the unloaded levels in each test scenario was further fitted, as displayed in Figure 8.

As can be seen from Figure 8, all three energies (i.e., *U^l^_i_*, *U^e^_i_*, and *U^d^_i_*) decreased with increasing *W_c_* and *D_φ_*. Meanwhile, the energy reduction value for increasing *D_φ_* from 15° to 30° was significantly larger than that for increasing *D_φ_* from 0° to 15°, which indicates that the energy reduction value gradually rises as the *D_φ_* increases. In addition, the *U^e^_i_* curve experienced a tendency to move away from the *U^l^_i_* curve with a gradually decreasing percentage as the *W_c_* and *D_φ_* of the specimen increased, whereas the *U^d^_i_* curve gradually moved closer to the *U^l^_i_* curve, with a resulting increase in the percentage. This suggests that the capacity to store energy of gently inclined layered shale decreased with increasing *W_c_* and *D_φ_*.

All three energies exhibit a nonlinear growth trend with the increased unloading levels. Fitting *U^l^_i_*, *U^e^_i_*, and *U^d^_i_* to the actual unloading stress levels, respectively, we found that the fitting based on the quadratic relationship was the best, and the correlation coefficients were all above 0.9. This indicates that all three energy evolution laws are characterized by obvious nonlinearities during the whole process of the loading-and-unloading test under uniaxial compression condition.

2. Determination of elastic compression energy storage coefficient

**Figure 9:**
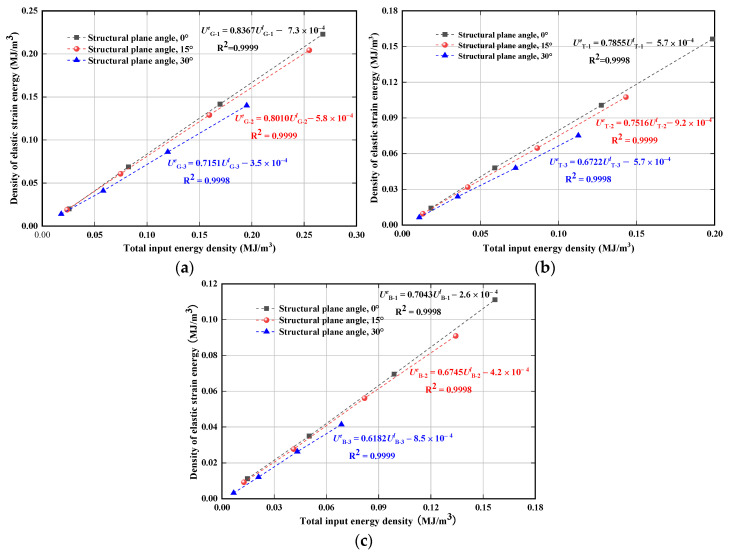
Curves of total input energy versus elastic energy for specimens under different test scenarios: (**a**) Dry; (**b**) Natural; (**c**) Saturated.

The *U^e^_i_* and *U^l^_i_* are selected for analysis to obtain the interrelationships among the energies at different unloading levels. To further investigate the ability of rocks to retain the *U^e^_i_* during energy evolution, the index of elastic compression energy storage *S* was introduced to characterize the relationship between the *U^e^_i_* and *U^l^_i_*. Figure 9 presents the variation patterns of the *U^e^_i_* and the *U^l^_i_* for each specimen at the different unloading levels. For the same specimen, the values of the *U^e^_i_* and *U^l^_i_* increased with the increase in the unloading level, and their distribution characteristics conformed to the linear growth law. The linear fitting results of elastic energy density and total input energy density of each specimen at various unloading levels are depicted as dashed lines in Figure 9. These two energies satisfied $U^{e}$ *= S*$U^{l}$ *+ a* under each test scenario, and the correlation coefficients were all above 0.9, indicating that *U^e^_i_* and *U^l^_i_* have a strong linear correlation in the loading-and-unloading test under the uniaxial compression condition. Considering that the value of the *a* is very small under each test scenario (three orders of magnitude smaller than *S*), the equation $U^{e}$ *= S*$U^{l}$ *+ a* can be simplified as $U^{e}$ *= S*$U^{l}$. The *S* is utilized to characterize the damage and crack development of the rock and its elastic energy storage capacity, and the larger the *S*, the higher the proportion of the stored elastic energy.

It should be noted that, due to the variations of the *D_φ_* and *W_c_*, there were some differences in the distribution patterns of the energy density results in each group of specimens. The *U^l^_i_* and *U^e^_i_* decreased with increasing *D_φ_* for the same *W_c_* and the unloading level, while the deformational energy-absorbing capacity of the rock specimens were weakened. The smaller the *D_φ_*, the larger the slope of the fitted straight line and the proportion of the stored elastic energy. For specimens with the same *D_φ_*, the slope of the fitted straight line decreased as *W_c_* increased, weakening the rock specimens’ capacity to store elastic energy. This further verified the water-softening and anisotropy properties of the gently inclined layered shale. It can be inferred that the *S* of the rock is determined by its own material characteristics and has no significant correlation with the unloading level. To further analyze the relationship between the *W_c_* and *D_φ_* and the *S*, the parameter variables (i.e., *W_c_* and *D_φ_*) and the test results (i.e., the *S*) for each group are summarized in Table 2.

Since unloading at the peak point cannot be realized in the actual test process, the relationships between the *W_c_* and *D_φ_* and the *S* need to be established first to determine the peak point elastic energy under uniaxial compression conditions. The *U^l^_i_* at the peak point can be calculated on the basis of the stress–strain curve under the conventional uniaxial compression conditions. Finally, the *U^e^_i_* at the peak point can be calculated based on equation $U^{e}$ *= S*$U^{l}$.

**Figure 10:**
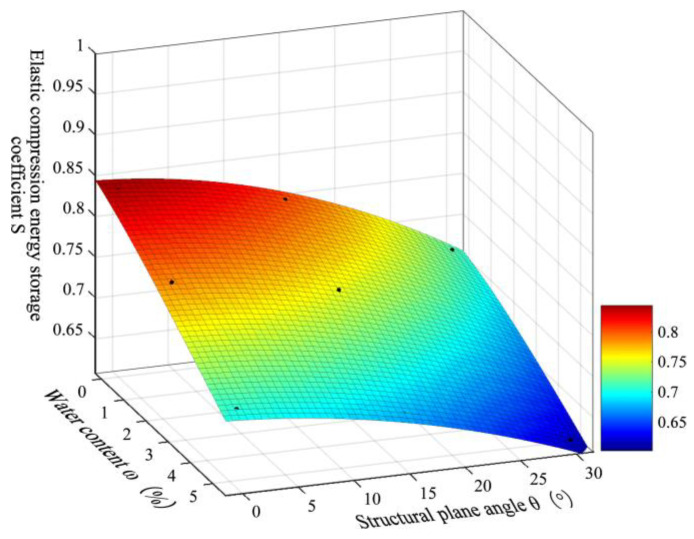
Relationship among the elastic compression energy storage coefficients (*S*), water content (*ω*), and structural plane angle (*θ*).

Analysis of Table 2 reveals that there is a specific relationship between the *S* of gently inclined layered shale and the *W_c_* and *D_φ_*, i.e., *S* = *f* (*W_c_*, *D_φ_*). The fitted surfaces with the statistical data of the *S* versus the *W_c_* and *D_φ_* in each group of specimens are presented in Figure 10. The relationship between the *W_c_* and *D_φ_* and the *S* of gently inclined layered shale from the Chaoyang Tunnel can be expressed as:

(5) $$S=\frac{0.8382+3.474E-02W_{c}-2.346E-03D_{\varphi }-9.977E-05D_{\varphi }{}^{2}}{1+6.399E-02W_{c}+3.975E-03W_{c}{}^{2}-1.668E-03D_{\varphi }}$$

The elastic energy at the peak point is:

(6) $$U_{peak}^{e}=SU_{peak}^{l}=\frac{0.8382+3.474E-02W_{c}-2.346E-03D_{\varphi }-9.977E-05D_{\varphi }{}^{2}}{1+6.399E-02W_{c}+3.975E-03W_{c}{}^{2}-1.668E-03D_{\varphi }}\cdot U_{peak}^{l}$$

### 4.3. Deformation Tendency Criterion of Tunnel Bottom

In this section, the brittle ductility index *C* is introduced and combined with the *S* to establish an energy evolution-based discriminating indicator for the large deformation tendency in shale tunnels.

#### 4.3.1. Brittle Ductility Index

The index *C* was employed to characterize the rock specimen’s damage, and it was calculated by the following equation:

(7) $$C=\frac{U_{peak}^{e}-U^{a}}{U_{peak}^{e}}=1-\frac{U^{a}}{U_{peak}^{e}}$$

where *U^e^_peak_* represents the peak point elastic energy calculated by Equation (6); *U^a^* represents the post-peak residual energy obtained by integrating the post-peak segment of the stress–strain curve during the process of the conventional uniaxial compression test.

The evolution of rock brittle damage to ductile damage is essentially the transformation from local brittle damage to overall damage [33]. When the *C* approaches 1, it indicates that the rock undergoes brittle damage. When the *C* is close to or even less than 0, the localized brittle damage within the rock is converted into overall damage, which is manifested as ductile damage.

The phenomenon observed in the test reveal that, under the uniaxial compression condition, the gently inclined layered shale specimens undergo “damage and weakening of the structural plane” before the peak stress and “localized brittle damage” after the peak stress. The *C* is significantly affected by the *W_c_* and *D_φ_*, which indicates that the *C* gradually decreases as the *W_c_* rises, and the damage pattern of the specimen progressively transforms from brittle to ductile. However, due to the overall large value of the *C* of the specimen, it still exhibits the localized brittle damage along the weakened structural surface. In addition, as the value of index *C* decreases, the damage distribution within the rock mass becomes more uniform, and the rock is more likely to undergo overall ductile damage. Therefore, the *C* can comprehensively reflect the uniformity of pre-peak damage distribution and post-peak damage behavior of rock specimens. In addition, the higher the degree of rock energy dissipation, the more uniform the distribution of pre-peak damage, and the post-peak damage pattern changes to the overall ductile damage pattern. At this time, the rock mass is prone to overall deformation, i.e., the necessary conditions for deformation of gently inclined layered shale are the high degree of pre-peak energy dissipation and the extensive post-peak damage.

It can be concluded from the characteristics of the *S* and *C* that the deformation of the rock mass of the tunnel in gently inclined layered shale strata is characterized by asymmetry, which is specifically reflected in the fact that the rock mass is dominated by the local brittle damage and supplemented by the overall deformation. The bottom deformation of the tunnel is dominated by shear misalignment or bending and rumbling, and damage usually occurs along the structural surface. On this basis, this study integrated the elastic energy storage capacity of rocks (assessing the extent of rock damage before reaching peak strength) and the mode of brittle-ductile failure (i.e., the manner and form of energy released after rock mass failure). As a result, an energy-based discriminant indicator *SC* (*SC* = *S* + *C* – *S* × *C*) was developed for evaluating the deformation trend of the tunnel bottom in the gently inclined layered shale formation of the Chaoyang Tunnel. The *SC* values of the gently inclined layered shale specimens under the different test scenarios are present in Table 3.

#### 4.3.2. Establishment and Validation of Bottom Deformation Grading Indicator

**Figure 11:**
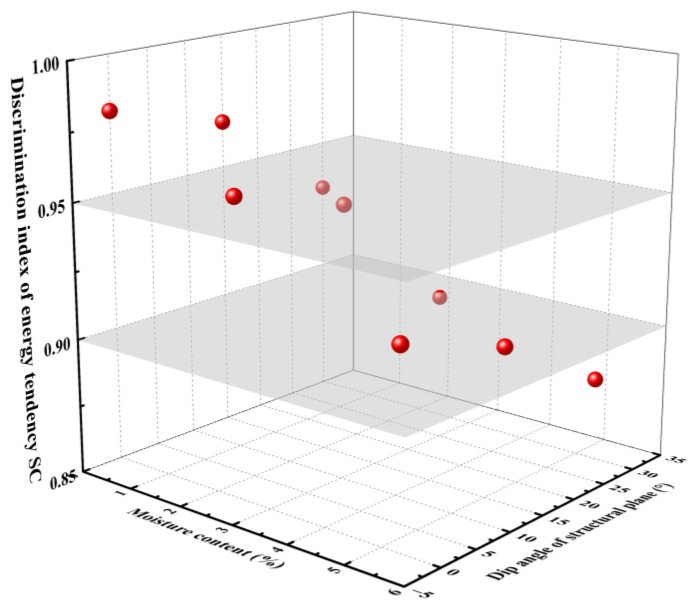
Three-dimensional distribution diagram of the *SC*.

During the actual construction process, influenced by the water-softening characteristics of the gently inclined layered shale and excavation disturbances, varying degrees of deformation occurred at the bottom of the Chao Yang Tunnel. This resulted in the cracking of the lining structure, posing a threat to the structural safety of the tunnel. To effectively assess the extent of tunnel deformation, the tunnel bottom deformation was classified based on the discriminant index *SC*. Figure 11 presents the relationships between the *W_c_* and the *SC*. It is observed that the *SC* is obviously distributed in a partition, which can be divided into four intervals of *SC* ≤ 0.85, 0.85 < *SC* ≤ 0.90, 0.90 < *SC* ≤ 0.95, and *SC* > 0.95. Table 4 shows the deformation grading criteria based on the *SC*.

**Figure 12:**
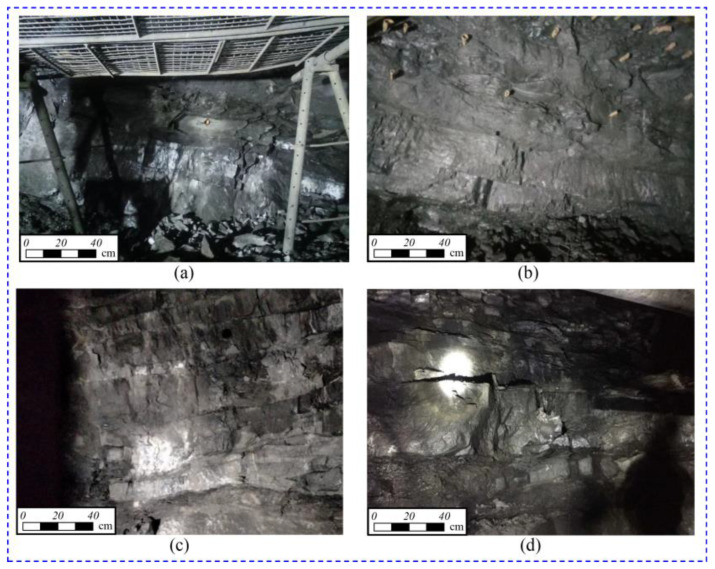
Surrounding rock condition of tunnel face: (**a**) DK 166+035; (**b**) DK166+145; (**c**) DK166+360; (**d**) DK166+440.

To verify the effectiveness of the proposed *SC*, this study compared the actual bottom deformation grades with the evaluation results acquired with the *SC*. The tunnel deformation monitoring on four sections was conducted with the use of the total station. The shale morphology at the excavated tunnel face is depicted in Figure 12. The tunnel faces at the construction site are composed of gently inclined layered shale. In Figure 12a, the layer thickness is relatively minimal, the dip angle is the highest, and the moisture content is the greatest, resulting in relatively fractured surrounding rock. In Figure 12c, the surrounding rock has the lowest moisture content, the smallest dip angle, and is relatively intact.

Some rock blocks are taken from the four selected sections to prepare the standard specimens, and conventional uniaxial compression tests were conducted. With the combination of the stress–strain curve obtained from the tests, the post-peak residual energy *U^a^* can be calculated, and then the *C* can be determined. Taking the section of DK166 + 035 as an example, the *D_φ_* is 26°, the *W_c_* is 5.42%, and the *S* can be calculated as 0.6323. Combined with the results of uniaxial compression test, the *C* can be obtained as 0.7041, and then a relationship of *SC* = 0.6323 + 0.7041 − 0.6323 × 0.7041 = 0.8912 can be determined. As can be seen from Table 4, the bottom deformation grade at the section of DK166 + 035 is grade III, with a medium degree of deformation, and the deformation value is between 300 and 500 mm, which matches with the onsite monitoring result (369 mm). Similarly, the *SC* values of the other three sections can be calculated with the monitoring results, as indicated in Table 5. The comparison of the field measurement results confirms that the prediction results based on the *SC* are consistent with the actual damage conditions of the rock mass, and this indicator can more accurately predict the bottom deformation tendency of the Chaoyang Tunnel in the gently inclined layered shale stratum.

For tunnels constructed in gently inclined layered shale formations, single-axis compression tests can be conducted onsite. The C-value and S-value are determined based on the stress–strain curve and rock sample characteristics (structural plane angle and moisture content). Subsequently, the tunnel bottom deformation discrimination index, the *SC* value, can be calculated, providing a prediction of tunnel bottom deformation. Based on the predicted deformation, reinforcement measures such as grouting, long anchor bolts and cables, optimizing invert curvature, and increasing invert thickness can be implemented. These measures aim to prevent significant tunnel deformations, ensuring structural safety.

## 5. Conclusions

By conducting conventional uniaxial compression tests and single-cycle loading–unloading uniaxial compression tests, the failure characteristics and energy evolution patterns of the gently inclined layered shale specimens with different *D_φ_* and *W_c_* were analyzed. Moreover, a discriminant indicator suitable for determining the deformation trend at the bottom of tunnels in layered shale was proposed. The conclusions obtained are as follows:

1. Water content and structural plane angle significantly affected the energy evolution characteristics of the shale specimens. The larger the *D_φ_*, the further the performance curve moved away from the *U^l^_i_* curve, and the closer the *U^d^_i_* curve approached the *U^l^_i_* curve, indicating that the proportion of elastic energy in the total input energy gradually decreased while the proportion of dissipated energy gradually increased. The increase in the *W_c_* led to the decrease in energy storage capacity of the shale specimen, which was manifested in a further aggravation of internal damage.
2. An increase in the moisture content and structural surface angle weakened the energy storage capacity of the layered and gently dipping shale. Under uniaxial compression conditions, the total input energy *U^l^_i_*, the elastic energy *U^e^_i_*, and the dissipated energy *U^d^_i_* exhibited quadratic relationships with the unloading level.
3. During the single-cyclic loading-and-unloading uniaxial compression tests, the *U^l^* and *U^e^* of the shale specimens exhibited a strong linear correlation, which was determined by the moisture content and the structural plane angle of the layered shale specimens, i.e., *U^e^ = f* (*w*, *β*) *U^l^*.
4. Based on the energy change characteristics of the shale specimens, a discriminant parameter SC was proposed for determining the deformation trend at the bottom of the tunnel. The tunnel bottom deformation was classified into four levels: no deformation, slight deformation, moderate deformation, and severe deformation. The proposed discriminant parameter SC can accurately predict the amount of tunnel bottom deformation, providing a reference for the safety assessment of actual construction when traversing through the gently inclined layered shale formations.

The results of this study have confirmed the feasibility of using energy index test val-ues under uniaxial compression conditions to predict the deformation of tunnel structures in shale formations. In future studies, the effects of wet and dry processes on the rock mass will be considered [34], as well as the effects of rock sample type and multidimensional stress state. The triaxial loading–unloading tests on various rock samples will be conducted to analyze the correlation between the energy characteristic values and the rock sample structural parameters under complex stress states. Given this, the rock layer thickness will be considered as an uncertainty factor and the uncertainty will be quantified using confidence intervals. The proposed method will be validated through established practices to demonstrate its feasibility. Then, the energy evolution characteristics of different rock samples under triaxial stress conditions will be elucidated. The aim is to establish a tunnel deformation prediction model and evaluation system based on real stress states, providing a more systematic reference for the safety assessment of tunnel construction.

## Figures and Tables

**Table 1:** Specimen working condition and number.

Specimen No.	Water Content (*W_c_*)	Dip Angle of Structural Plane (*D_φ_*)	Unloading Levels
G-1-i	Dry (0%)	0°	*i* = 20%, 40%, 60%, 80%
G-2-i		15°	
G-3-i		30°	
T-1-i	Natural (2.4%)	0°	
T-2-i		15°	
T-3-i		30°	
B-1-i	Saturated (5.3%)	0°	
B-2-i		15°	
B-3-i		30°	

**Table 2:** Statistics of the S values of gently inclined layered shale under different test scenarios.

Specimen No.	Water Content (*W_c_*)	Dip Angle of Structural Plane (*D_φ_*)	Elastic Compression Energy Storage Coefficients *S*
G-1	0%	0°	0.8367
G-2	0%	15°	0.8010
G-3	0%	30°	0.7151
T-1	2.4%	0°	0.7855
T-2	2.4%	15°	0.7516
T-3	2.4%	30°	0.6722
B-1	5.3%	0°	0.7043
B-2	5.3%	15°	0.6745
B-3	5.3%	30°	0.6182

**Table 3:** Statistical table of *SC* value for gently sloping layered shale specimens.

Specimen No.	Elastic Compression Energy Storage Coefficients (*S*)	Brittle Ductility Index (*C*)	Indicators of Tunnel Bottom Deformation Tendency (*SC*)
G-1	0.8367	0.8763	0.9798
G-2	0.8010	0.8345	0.9671
G-3	0.7151	0.7601	0.9317
T-1	0.7855	0.8072	0.9586
T-2	0.7516	0.7791	0.9451
T-3	0.6722	0.6879	0.8977
B-1	0.7043	0.7358	0.9219
B-2	0.6745	0.7147	0.9071
B-3	0.6182	0.6874	0.8806

**Table 4:** Bottom deformation grading criteria based on the *SC*.

*SC* Value	Bottom Deformation Grade	Bottom Deformation (mm)
*SC* > 0.95	I (no deformation)	<100
0.95 < *SC* ≤ 0.90	II (slight deformation)	100~300
0.90 < *SC* ≤ 0.85	III (moderate deformation)	300~500
*SC <* 0.85	IV (severe deformation)	>500

**Table 5:** Bottom deformation grades at different sections of the Chaoyang Tunnel.

Section Stake Number	Description of the Basic Conditions	Predicted Values of the Tunnel Bottom Deformation (mm)	Monitoring Values of the Tunnel Bottom Deformation (mm)	*SC* Value	Determination of the Tunnel Bottom Deformation Degree
DK166 + 035	Structural plane angle of 26°, saturated	300~500	369	0.8912 (Grade III)	Moderate deformation
DK166 + 145	Structural plane angle of 21°, natural	100~300	241	0.9139 (Grade II)	Slight deformation
DK166 + 360	Structural plane angle of 11°, natural	<100	91	0.9517 (Grade I)	No deformation
DK166 + 440	Structural plane angle of 14°, natural	100~300	152	0.9493 (Grade II)	Slight deformation

## Data Availability

Data are contained within the article.
